# Phenotypic factor analysis of psychopathology reveals a new body-related transdiagnostic factor

**DOI:** 10.1371/journal.pone.0177674

**Published:** 2017-05-18

**Authors:** Patrizia Pezzoli, Jan Antfolk, Pekka Santtila

**Affiliations:** 1 Department of Psychology, Åbo Akademi University, Turku, Finland; 2 Turku Brain and Mind Centre, Turku, Finland; United (Osaka U, Kanazawa U, Hamamatsu U Sch Med, Chiba U and Fukui U) Graduate School of Child Developmen, JAPAN

## Abstract

Comorbidity challenges the notion of mental disorders as discrete categories. An increasing body of literature shows that symptoms cut across traditional diagnostic boundaries and interact in shaping the latent structure of psychopathology. Using exploratory and confirmatory factor analysis, we reveal the latent sources of covariation among nine measures of psychopathological functioning in a population-based sample of 13024 Finnish twins and their siblings. By implementing unidimensional, multidimensional, second-order, and bifactor models, we illustrate the relationships between observed variables, specific, and general latent factors. We also provide the first investigation to date of measurement invariance of the bifactor model of psychopathology across gender and age groups. Our main result is the identification of a distinct “Body” factor, alongside the previously identified Internalizing and Externalizing factors. We also report relevant cross-disorder associations, especially between body-related psychopathology and trait anger, as well as substantial sex and age differences in observed and latent means. The findings expand the meta-structure of psychopathology, with implications for empirical and clinical practice, and demonstrate shared mechanisms underlying attitudes towards nutrition, self-image, sexuality and anger, with gender- and age-specific features.

## Introduction

### Latent variable models of psychopathology

Comorbidity, the coexistence of two or more psychopathological conditions, represents a persistent challenge to the representation of mental disorders as discrete categories historically implied by diagnostic systems [1, 2]. Multiple studies have attempted to describe the sources of covariation between disorders in terms of categorical, dimensional, and hybrid latent structures [3–6]. In most cases, factor analytic approaches provided a better-fitting account of comorbidity. Factor analysis estimates the association between observed variables, referred to as indicators or items, and fewer underlying dimensions, named factors or constructs. Latent variable modeling procedures have repeatedly indicated the existence of two latent dimensions of psychopathology: Internalizing and Externalizing [7–28]. The Internalizing construct represents a tendency to introject distress, and has been conceptualized either as a unitary dimension or bifurcating into the lower-order subcomponents of Distress and Fear [11]. The Externalizing construct describes the propensity to express distress in outward-directed actions and includes substance use disorders (e.g., substance abuse/dependence) and behavioral problems (e.g., conduct disorder, oppositional defiant disorder, and anti-social personality disorder). These two factors have been shown to be under genetic control and to account for the coexistence and continuity over time of putatively distinct disorders [13]. The Internalizing-Externalizing meta-structure has been replicated in populations from across the world [16] and has been shown to be gender invariant [25, 26]. The replicated observation that the Internalizing and Externalizing constructs are correlated gave rise to the appealing hypothesis of a general factor of psychopathology, or “P” factor. This hypothesis has received support from studies employing bifactor models, which accommodate a general factor in addition to symptom-specific domains [27–29]. In these models, the Internalizing and Externalizing constructs explain a proportion of common variance for their corresponding indicators, while the overarching “P” factor explains the proportion of variance common to all available indicators. These models allow researchers to study the relationship between observed variables and their specific factors, independently of a common latent dimension, and to compare mean levels of general and specific factors across groups. Bifactor models of psychopathology are gaining increasing traction for their ability to incorporate multidimensionality, outperforming correlated-factor and unidimensional models. However, they seem to provide a better account of psychological functioning for statistical rather than theoretical reasons, specifically by accommodating more measurement noise [30, 31]. As a consequence, their interpretation remains controversial, and conclusions on the best-fitting bifactor solution should not discourage examination of alternative models.

### Expanding cross-disorder research

Most of the existing structural research has focused on common mental disorders, overlooking diagnoses that have lower prevalence rates. When incorporated, some of these disorders reflect sub-components of the Internalizing and Externalizing constructs, while others appear as additional factors. For instance, previous studies included psychotic disorders and observed that they load onto a unique “Thought Disorder” construct [7, 28, 32, 33]. It has also been proposed to include personality traits, historically considered separately from psychiatric disorders, into a coherent spectrum of individual differences [33–36]. Of particular interest to the present study are anger and aggressive behavior, feelings related to sexual life, quality of body image and eating attitudes. Disinhibitory traits like aggression and impulsivity have been extensively correlated with externalizing forms of psychopathology. Although impulse control disorders share similar etiologic and neuropsychological correlates with externalizing disorders, their comorbidity with internalizing psychopathology is often reported [19, 37–40]. Only a few studies have explored the inclusion of impulse control disorders within a factorial structure alongside other psychiatric disorders and confirmed their inclusion in the broader Externalizing spectrum [19, 33, 40]. Notably, intermittent explosive disorder has been reported to cross-load on Internalizing, particularly on the Fear sub-factor, suggesting that anxious avoidance and anger might be complimentary or co-occurring responses in situations of increased negative affect [19]. Sexual dysfunctions also show high rates of comorbidity with internalizing disorders, denoting a plausibly common underlying psychopathological etiology. Epidemiological surveys report high prevalence rates of sexual dysfunction symptoms, strongly associated with poor quality of life, poor mental health, negative affect, low self-esteem and interpersonal function [41]. To our best knowledge, only one research group has previously included measures of sexual dysfunctions in the broad Internalizing-Externalizing framework [41–43]. The results of their studies support a hierarchical three-factor model, with a higher-order Internalizing construct and three (Distress, Fear and Sexual Problems) sub-constructs. Until recently, symptoms associated with disordered eating and body image have also been neglected, despite their comorbidity with disorders like depression, anxiety and substance use [44]. Consecutive studies [19, 45, 46] described best-fitting models where eating disorders represented a sub-construct within the Internalizing spectrum, compared to models where they defined their own diagnostic construct, loaded onto Externalizing, or cross-loaded onto both. Only one study, to our knowledge, conducted multi-group measurement invariance of this best-fitting structure and supported its statistical consistency across women and men [46].

### The present study

In sum, the present study was aimed at expanding the Internalizing- Externalizing meta-structure of psychopathology, at testing alternative dimensional conceptualizations of comorbidity, and at providing the first measurement invariance test of the bifactor model of psychopathology. We hypothesized that new latent dimensions would originate from the inclusion and combination of cross-disorder symptoms measured in the general population, and that comparing different structural models would reveal stable associations, within and between latent dimensions. Our results supported these hypotheses. We further predicted that gender and age differences in psychological functioning would be explained by differences in mean factor levels. In partial contrast with our prediction, measurement invariance was only established across generations, allowing for meaningful group comparisons on the latent means.

## Materials and methods

### Sample

The 13024 individuals included in the present study (8415 women and 4609 men, age range 18–49) were recruited through the Central Population Registry of Finland during two major population-based data collections in 2005 and 2006, as part of the Genetics of Sexuality and Aggression (GSA) project [47], launched by the Åbo Akademi University in Turku, Finland. The research plans for both major data collections were approved by the Ethics Committee of the Åbo Akademi University, in accordance with the 1964 Declaration of Helsinki. A detailed description of the baseline demographic characteristics of our participants and the analysis of their representativeness of the population is provided elsewhere [47].

### Measures

Alongside dimensional measures of mental disorders that have been empirically shown to correspond to Internalizing and Externalizing liability dimensions, namely measures of anxiety and depression, psychopathy and alcohol use, we also included measures of indicators often neglected: trait anger and aggressive behaviour, sexual distress, eating attitudes and body image. Levels of depression and anxiety were measured using the Depression and Anxiety subscales of the Brief Symptom Inventory [48]. Alcohol use was assessed with the Alcohol Use Disorder Identification Test [49] and psychopathy with the Self-Report Psychopathy Lifestyle, Interpersonal, Antisocial and Affective subscales [50]. Levels of aggression were measured using the Physical and Verbal subscales of the Aggression Questionnaire [51]; trait anger with the trait anger subscale of the State Trait Anger Expression Inventory II [52]; sexual distress with the gender-neutral items of the Female Sexual Distress scale [53]. We assessed eating attitudes with the Eating Attitudes Test [54] and body image using the Body Image scale of the Derogatis Sexual Functioning Inventory [55]. All scales consisted of Likert-type ratings. Inspection of the response distribution suggested that items could be handled as continuous measurements.

### Statistical analyses

Given that substantial evidence favors dimensional models of psychopathology, we used factor analysis to explore the structure underlying our data. As most statistical methods, factor analysis assumes that the available sample consists of independent observations. However, in our case, indicators were measured on individuals nested within families. Twins and siblings of twins partly share environment and genetic make-up, they interact and might be exposed to or seek out similar experiences. Consequently, data nested within families cannot be considered independent. When no correction is used for phenotypic analysis of family data, standard errors and goodness-of fit tests are biased. Based on these considerations, we implemented complex sampling correction by treating each family as a cluster variable and estimating model parameters aggregated over clusters, with standard errors and scaled test statistics that are robust to both non-normality and non-independence of observations [56].

Analyses were performed using SPSS Statistics for Macintosh, version 23.0 [57] and in R environment for statistical computing, version 3.3.2 [58], using the packages data.table [59], nFactors [60], psy [61], lavaan.survey [62], semTools [63] and semPlot [64].

#### Exploratory factor analysis (EFA)

Starting from individual items, we created composite scores to be included in subsequent measurement models. This was done using a maximum likelihood EFA, extracting a single factor and saving the score derived using the Bartlett method. Internal consistency of the composite scores was good overall (Cronbach’s alpha: Depression = .85; Anxiety = .85; Sexual distress = .90; Trait anger = .85; Alcohol use = .86; Aggression = .85; Psychopathy = .80; Eating attitudes = .84; Body image = .72). Extreme multicollinearity and singularity could be excluded, since no correlation between the indicators was larger than .68. A second EFA was performed on the composite variables to determine the number of underlying factors. This determination was based on visual inspection of the Kaiser-Guttman and Cattel scree plots. To identify the hierarchical structure of our data, we performed exploratory bifactoring by calculating the indirect effect of a broader construct on the indicators through the specific correlated factors (second-order structure), as well as its direct effect (bifactor structure). The higher-order construct was extracted from the already identified correlated-factor solution. With Schmid-Leiman orthogonalization, the second-order solution was transformed to allow all measured variables to load on any number of specific factors and, at the same time, on the general construct [65].

#### Confirmatory factor analysis (CFA)

Measurement and structural models were specified based on the exploratory results and tested against the alternative potential explanations for the common variance in our set of indicators. We assigned a scale to the latent constructs in our measurement models by fixing their variance to one, thus standardizing the factor loadings. When we were interested in freely estimating factor variances, for example across groups, we adopted the alternative “marker variable” strategy, fixing the loading of one indicator per factor to 1. Scale reliability was assessed using the omega and omega hierarchical coefficients [66], convergent validity by calculating the average variance extracted (AVE) of each factor, discriminant validity by assuring that the squared AVE was larger than the highest factor correlation. Model refinement was performed by incorporating statistically and theoretically reasonable modifications. No indicator was dropped based on its standardized squared loading when validity and fit indices supported the model. When unique indicator variances were smaller than a positive lower bound, but basic identification conditions were satisfied and convergence obtained, we employed constrained estimation to a positive value near zero (.1), reducing the bias in parameter estimates [67]. Cross-loadings were based on theoretical rationale, on the EFA pattern matrices and on model fit modification indices. Covariance of the error terms of the manifest variables was allowed according to meaningfulness, transitivity and generality rules [68]. We employed a combination of tests of absolute and relative fit to describe whether the proposed models fitted the data. Among the indices of global model fit, we report the Goodness of Fit Index (GFI) [69], which assumes values between 0 and 1, the latter indicating exact fit. We also report the Root Mean Square Error of Approximation (RMSEA), a measure of the estimated discrepancy between the population and the model-implied covariance matrices per degrees of freedom [70]. RMSEA values larger than .06 are generally considered as indicative of good fit, values close to .08 as indicative of mediocre fit [71], therefore we chose a stringent cut-off value of .07 [72]. The likelihood ratio chi-square test is also presented. However, this test rejects reasonable models when the sample size is large, like in the present study, so we did not rely upon this statistic as a basis for model acceptance [72]. To choose among competing models with a different number of parameters, we used two information-theoretic measures, the Akaike Information Criterion (AIC) [73] and the Bayesian Information Criterion (BIC) [74]. In both cases, lower values indicate a better tradeoff between model fit and model complexity.

#### Tests for invariance

To clarify whether the identified best-fitting factor structure was equivalent across gender and age in our sample, measurement and strict invariance tests were performed. Specifically, we conducted a series of nested model comparisons, where each step contained additional restrictions (equality constraints). First, we tested if the same factor structure was valid in each group by running configural models (i.e., baseline multi-group models with no equality constraints). After this, we tested metric models by constraining the factor loadings to be equal across groups. Once we had established invariance of the unit of measurement, we tested scalar (or “strong”) invariance, which is required to compare groups’ mean scores on the latent variables. In a scalar invariance model, all intercepts, representing the origin, or starting value, of the indicators, are constrained to be equal across groups [75]. When this stringent level of invariance could not be established, we tested partial scalar invariance to understand which intercepts differed across groups. [76]. To compare group means we performed a structured means analysis [77]. The rationale behind latent mean invariance test is that observed mean differences do not necessarily indicate latent mean differences, because observed indicators’ means are also a function of their intercepts and loadings. When latent means comparisons were precluded, we only reported observed differences. Finally, strict invariance, or full uniqueness, was investigated with constrained indicator variance and covariance as well as factor variance and covariance invariance models. When comparing nested models and assessing adequacy of the restrictions, we complied with the Cheung–Rensvold criteria, or ΔCFI-rule, with differences greater than or equal to .01 in the Comparative Fit Index (CFI) supporting non-invariance [78, 79]. We also relied on a combination of two global fit indices: the RMSEA and the standardized root mean square residual (SRMR) [80]. Invariance was discarded with changes in RMSEA ≥ .01, supplemented by changes in SRMR ≥ .03 (when testing metric models) or ≥ .01 (with scalar models).

## Results

### The latent structure of psychopathology in our sample

Inspection of the scree plots indicated the presence of three factors accounting for the observed pattern of scores. Exploratory factor analysis with promax rotation extracted three factors. Measures of psychopathy, aggression, trait anger and alcohol use were indicative of a first factor, defined as the Externalizing construct (factor loadings: .88, .76, .50, .48, respectively). Measures of depression, anxiety and sexual distress loaded on a second, Internalizing construct (factor loadings: .99, .76, .38). Measures of eating attitudes and body image created a third factor, here referred to as the Body construct (factor loadings: .88, .76). This solution explained 56.6% of the variance. Of note, two indicators cross-loaded onto the Body factor, namely sexual distress (.21) and trait anger (.28). We explored an alternative two-factor solution, consistent with the Internalizing-Externalizing structure of psychopathology. When two factors were extracted, measures of depression, anxiety, sexual distress, body image and eating attitudes loaded on the Internalizing factor (factor loadings: .81, .71, .56, .52, .52, respectively), measures of psychopathy, aggression, alcohol use and trait anger loaded on the Externalizing factor (factor loadings: .81, .77, .53, .39). This solution explained 43.4% of the variance. To test the hypothesis of a unidimensional factor structure, one factor score was extracted from all the available measures of adult psychopathology, but this solution explained only 28.1% of the variance. We then tested whether a hierarchical structure would better capture the multidimensional nature of our data, while uncovering a single common “P” factor. In the second-order model, a common source of variation between first-order factors was included and, in the bifactor model, direct connections between indicators and all latent factors (common and specific) were modeled. The total variance explained was almost evenly distributed across general and specific factors, as indicated by the Explained Common Variance (ECV = .44) and the Omega Hierarchical reliability index (ωH = .54) [66].

**Fig 1:**
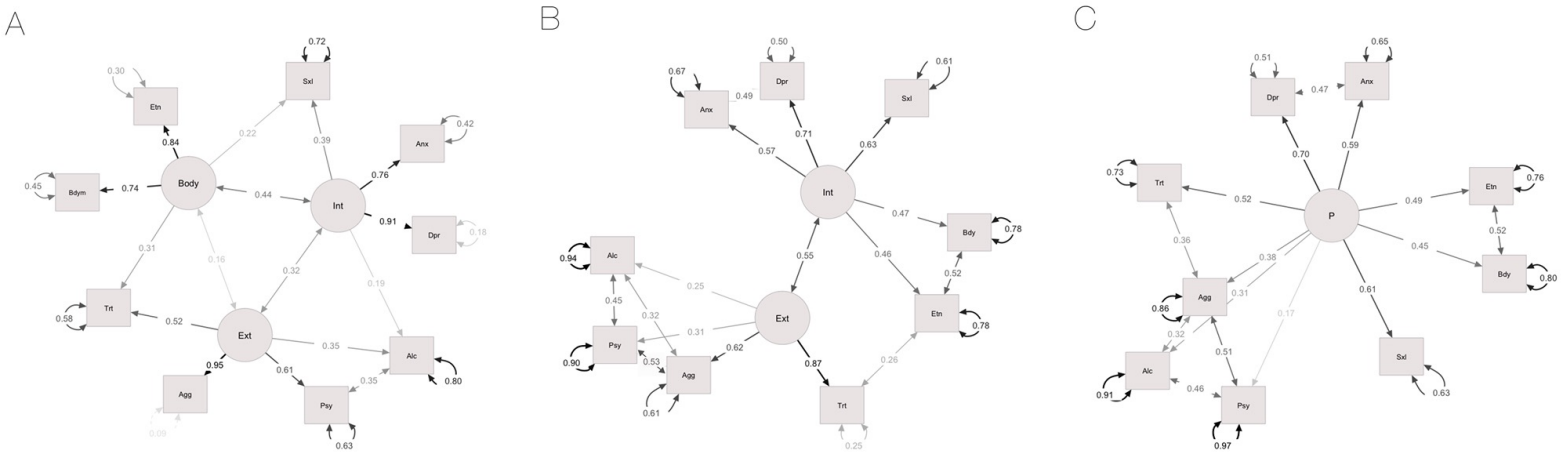
Correlated-factor models. 1A: three-factor model (Model 1); 1B: two-factor model (Model 2); 1C: one-factor model (Model 3). Int = Internalizing; Ext = Externalizing; Dpr = Depression; Anx = Anxiety; Agg = Aggression; Psy = Psychopathy; Alc = Alcohol use; Trt = Trait anger; Bdym = Body image; Etn = Eating attitudes; Sxl = Sexual distress. Rectangles represent manifest variables, circles represent latent variables. Residual variances of the manifest variables are reported on curved lines with double-headed arrows. Residual variances of the latent factors were set to unity and are not reported here. Straight lines with double-headed arrows connecting the residuals of two variables indicate non-causal associations. Factor loadings are reported on straight lines with single-headed arrows, manifest variables are at the head of arrow.

Once the number of factors and their correspondence with the indicators were clarified, we analyzed the measurement models presented in Fig 1 with CFA. First, we analyzed the correlated (oblique) three-factor structure presented in Fig 1A (Model 1). Since the Body construct had only two indicators, we expected it to borrow information from other constructs. As predicted, and consistent with the exploratory results, sexual distress and trait anger cross-loaded onto it. Alcohol use showed an R^2^ value smaller than .4 in the unadjusted model (.24) and modification indices indicated that its correlation with psychopathy and its cross-loading onto Internalizing were associated with a large expected parameter change. Acceptable model fit was reached with these adjustments. We then tested the alternative two-factor solution displayed in Fig 1B (Model 2). Acceptable fit was obtained when several residuals were allowed to correlate within each factor as well as between trait anger and eating attitudes. We also tested the one-factor model presented in Fig 1C (Model 3) and established acceptable fit when, in addition to the pattern of correlated residuals specified for the two-factor model, we allowed aggression and trait anger to correlate. We concluded that the two- and one- factor models were miss-specified, based on the validity measures and on the observation that correlated measures were strongly influenced by the same latent variable, in addition to the one specified.

**Fig 2:**
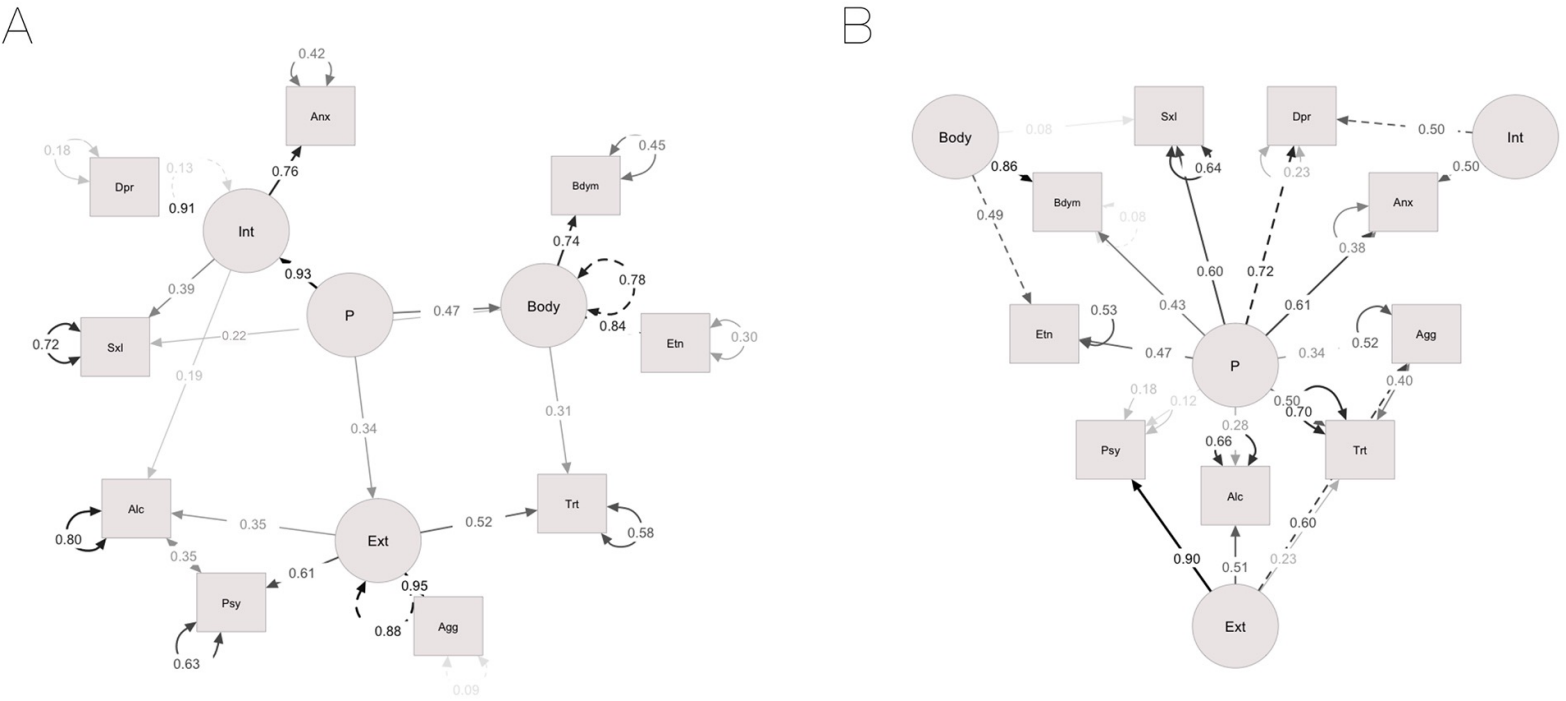
Hierarchical solutions with standardized factor loadings, residual variances and covariances. 2A: second-order model (Model 4); 2B: orthogonal bifactor model (Model 5). Int = Internalizing; Ext = Externalizing; Dpr = Depression; Anx = Anxiety; Agg = Aggression; Psy = Psychopathy; Alc = Alcohol use; Trt = Trait anger; Bdym = Body image; Etn = Eating attitudes; Sxl = Sexual distress. Rectangles represent manifest variables, circles represent latent variables. Residual variances of the manifest and first-order latent variables are reported on curved lines with double-headed arrows. The residual variance of the “P” factor was set to unity and is not reported here. Straight lines with double-headed arrows connecting the residuals of two variables indicate non-causal associations. Factor loadings are reported on straight lines with single-headed arrows, manifest and first-order latent variables are at the head of arrow.

The hierarchical structures tested with CFA are presented in Fig 2. To understand whether a second-order “P” factor would account for the estimated covariance among the first-order factors, we fitted a second-order model (Model 4, Fig 2A). First-order residuals described the variance in Internalizing, Externalizing and Body unexplained by the “P” factor (.08, .73 and .56, respectively). Baseline fit measures coincided with those of the oblique three-factor model, because the two hold the same number of free parameters. Likewise, refinement was needed to gain adequate fit. Compared to the oblique three-factor model, only the standardized variance of Internalizing was substantially reduced, suggesting that general psychopathology explained most of the variance in this factor (Model 1: Internalizing = .93, Externalizing = .83, Body = .74, Model 4: Internalizing = .12, Externalizing = .84, Body = .77). To estimate how much of the variance in the indicators could be explained by the “P” factor, beyond the unique contribution of the specific factors, we fitted a bifactor model (Model 5, Fig 2B). Here, the “P” factor represented individual differences on a common dimension. The remaining orthogonal constructs represented “group” or “nuisance” factors capturing the variance not accounted for by the “P” factor. Notably, in exploratory bifactoring, sexual distress loaded more strongly onto Body (.20) than onto Internalizing (.07) and was consequently included in CFA as an indicator of the Body construct. Acceptable fit was reached by allowing the correlation between the error terms of aggression and trait anger. Variance in depression, sexual distress and trait anger was especially described by the “P” factor, onto which these indicators loaded more strongly (.66, .57, .58) than on their specific factor (.57, .08, .33). Model 5 was compared with another bifactor model, where the general factor loadings were constrained to zero (Model 6). Regression weights associated with alcohol use and psychopathy were larger in Model 5 (.51, .81, respectively) compared to Model 6 (.46 and .64), indicating that those indicators were more indicative of externalizing manifestations than a broader psychopathology dimension. All the remaining regression weights were larger in Model 6, suggesting that a general psychopathology construct accounted well for variation in those traits. To quantify how the correlations between Internalizing, Externalizing and Body changed with the inclusion of a “P” factor in the model, we also fitted an oblique bifactor model (Model 7) and tested it against a specular oblique three-factor model (Model 8). The correlation between Internalizing and Externalizing was similar in the two models (three-factor = .39, bifactor = .41). Conversely, Body was positively correlated with Internalizing (.61) and Externalizing (.29) in the three-factor model, but these correlations became negative in the bifactor (-.23 and -.38, respectively). This result indicates that levels on a common latent dimension might be responsible for the covariance of body-related problems with multiple forms of psychopathology.

In order to decide which of the described models fitted our data best, we examined the fit indices of the baseline (unadjusted) models, reported in Table 1 alongside reliability and validity indices. Consistently with the EFA results, the oblique three-factor fitted our data better than the competing first-order models. Among hierarchical models, the bifactor model showed the best fit and, overall, best explained the pattern of results in our population.

**Table 1:** Reliability, validity and fit indices of the confirmatory factor models.

	Omega (total)	AVE (total)	χ^2^	df	*p*	GFI	RMSEA (90% CI)	AIC	BIC
Baseline									
**Model 1**	0.80	0.51	4968.28	24	<0.001	0.92	0.13	314802.10	315026.34
**Model 2**	0.76	0.41	9908.56	26	<0.001	0.86	0.17	319738.39	319947.67
**Model 3**	0.73	0.28	17970.95	27	<0.001	0.76	0.23	327798.77	328000.59
**Model 4**	0.80	0.52	4968.28	24	<0.001	0.92	0.13	314802.10	315026.34
**Model 5**	0.87	0.58	2680.03	20	<0.001	0.95	0.10	312521.86	312775.99
Adjusted									
**Model 1**	0.86	0.54	1348.39	21	<0.001	0.98	0.070	311188.22	311434.88
**Model 2**	0.65	0.33	1362.28	20	<0.001	0.98	0.072	311204.11	311458.24
**Model 3**	0.60	0.24	1631.13	20	<0.001	0.97	0.079	311472.96	311727.09
**Model 4**	0.86	0.54	1348.39	21	<0.001	0.98	0.070	311188.22	311434.88
**Model 5**	0.86	0.57	1287.13	19	<0.001	0.98	0.072	311130.96	311392.57
**Model 6**	0.47	0.52	9675.44	28	<0.001	0.85	0.16	319501.26	319695.60
**Model 7**	0.85	0.58	2302.98	16	<0.001	0.96	0.11	312152.81	312436.84
**Model 8**	0.87	0.57	8265.42	27	<0.001	0.88	0.15	318093.24	318295.06

Omega = coefficients omega; AVE = average variance extracted; χ^2^ = chi-square test; df = degrees of freedom; p = p-value associated with the chi-square test; GFI = Goodness of Fit Index; RMSEA = Root Mean Square Error of Approximation; CI = Confidence Interval; AIC = Akaike Information Criterion; BIC = Bayesian Information Criterion. Models: 1 = oblique three-factor; 2 = oblique two-factor; 3 = one-factor; 4 = second-order; 5 = bifactor; 6 = constrained bifactor; 7 = oblique bifactor; 8 = oblique three-factor with sexual distress indicator loading onto Internalizing.

### Gender and age differences

We then examined the consistency of our best-fitting orthogonal bifactor model across gender and age.

To test measurement invariance across women and men, we first specified a multi-group configural model to serve as a baseline for subsequent restrictions on the parameters (Configural 1). Allowing estimates to differ across groups resulted in improved fit. The two metric models, constraining the regression weights of the indicators on their nuisance factors (Metric 1), as well as their regression weights on the general “P” factor (Metric 2), could be accepted. We then tested a first scalar model by constraining, the intercepts of the indicators (Scalar 1). The combination of fit indices implied a significant deterioration in fit. A partial scalar invariance model was derived by releasing the intercepts one by one, starting from those with the largest group differences, in order to identify the ones responsible for non-invariance. A moderate improvement in fit was noticed in partial invariance models where alcohol use, trait anger, eating attitudes and psychopathy were separately released, but the only significant improvement was associated with the release of sexual distress (Partial scalar 1). When the intercepts of the nuisance factors were constrained equal, in addition to all but sexual distress’ intercept, fit significantly worsened and partial invariance testing did not improve it (Scalar 2). For completeness, we also carried out measurement invariance testing of the correlated three-factor model, but metric invariance was, again, the highest level of invariance established. Group comparisons on the latent means can be inaccurate without full intercept invariance [81], therefore we can only report mean differences on the observed variables. Women presented the highest levels of all the internalizing and, especially, body-related problems compared to men who, in contrast, had higher standing on the externalizing measures. Trait anger represented the only notable exception, with women reporting higher levels. Lastly, we tested strict invariance to examine gender differences in the residual variance and covariance of the observed and nuisance variables. The residual variance invariance models of indicators (Residual 1) and nuisance factors (Residual 2) were nested within the full metric model (Metric 2), while an oblique full metric model was specified for covariance invariance tests (Metric 3). Residual variances of the nuisance factors were invariant, residual variances of the observed variables were not. Similarly, a model freely estimating all the covariances of the indicators and constraining them equal across groups hardly converged, fitted very poorly and, not surprisingly, significantly worse than its baseline (Covariance 1), while covariance invariance was reached at the nuisance factors level (Covariance 2).

We then divided our sample into three groups to assess measurement invariance across generations. The first group comprised young adults, 18 to 25 years of age (N = 4484) and the second group included adults 26 to 32 years old (N = 4236). Individuals aged 33 to 49 (N = 4304) were included in a third group and are referred to as middle-aged. The same method and criteria described above for gender tests was adopted. The baseline configural bifactor model (Configural 2) showed comparable fit to the orthogonal bifactor. We established invariance of the regression weights on the nuisance and general factors (Metric 4 and 5, respectively), as well as invariance of the intercepts of indicators and nuisance factors (Scalar 3 and 4) and therefore could compare group means. No substantial change in fit was observed when the latent means on the three specific factors were constrained to equality across groups (Means 1), compared to the full scalar model, where factor means are fixed to zero in the first group so that intercepts can be estimated (Scalar 4). To compare mean levels of internalizing, externalizing and body-related psychopathology across groups, a series of factor-level tests was carried out. In each test, two groups were chosen as reference groups and their mean on one factor was fixed to zero, allowing the mean of the remaining group to differ. This unconstrained mean was not estimated in absolute sense, rather it reflected standardized latent mean differences across groups. The group of young adults showed the highest levels on the Internalizing construct, middle-aged individuals showed the lowest. The opposite pattern was observed on the Body factor. Young adults also had higher standing on the Externalizing and “P” factors, compared to adults and middle-aged participants. The effect size of the magnitude of these differences was medium when the means of groups 1 and 3 on Internalizing were freely estimated, small in the remaining comparisons. To test strict invariance, residual indicator and factor variance invariance models (Residual 3 and 4, respectively) and residual indicator and factor covariance invariance models (Covariance 3 and 4, respectively) were nested within the full scalar model. An oblique full scalar model was specified to serve as the baseline for covariance invariance tests (Scalar 5). Strict invariance did not hold for indicator variances and covariances. The amount of indicator variance unexplained by the latent traits (neither by the specific, nor by the common factor) is very likely to reflect measurement error, so it was unsurprisingly inequivalent across groups. Similarly, a model forcing equivalent indicator covariance, inconsistent with the nature of the bifactor model where group and common factors “compete” for explaining indicator covariance, did not converge. Notwithstanding, factor uniquenesses, which synthesize the common content of their indicators, as well as their covariances, showed group invariance. Model fit indices of the measurement and strict invariance tests performed across gender and age groups are presented in Table 2.

**Table 2:** Fit indices of the multi-group nested models.

Model	CFI	RMSEA	SRMR	Δ CFI	Δ RMSEA	Δ SRMR
Gender MI						
**Configural 1**	0.95	0.083	0.035			
**Metric 1**	0.95	0.092	0.038	-0.005	-0.009	-0.003
**Metric 2**	0.94	0.087	0.045	-0.005	-0.005	-0.007
**Scalar 1**	0.91	0.10	0.063	-0.034	-0.013	-0.018
**Partial scalar 1**	0.93	0.088	0.052	-0.013	-0.001	-0.007
**Scalar 2**	0.84	0.13	0.12	-0.092	-0.041	-0.067
**Residual 1**	0.92	0.094	0.060	-0.023	-0.007	-0.015
**Residual 2**	0.93	0.090	0.060	-0.008	-0.003	-0.015
**Metric 3**	0.94	0.098	0.044	-	-	-
**Covariance 1**	0.92	0.18	0.22	-0.019	-0.080	-0.17
**Covariance 2**	0.94	0.094	0.044	0.000	-0.004	0.000
Age MI						
**Configural 2**	0.93	0.10	0.045			
**Metric 4**	0.93	0.11	0.046	-0.001	-0.003	-0.001
**Metric 5**	0.93	0.096	0.050	-0.001	0.011	-0.004
**Scalar 3**	0.93	0.088	0.052	-0.006	0.008	-0.002
**Scalar 4**	0.92	0.088	0.055	-0.006	0.000	-0.003
**Means 1**	0.92	0.089	0.061	-0.002	-0.001	-0.006
**Residual 3**	0.77	0.14	0.096	-0.15	-0.052	-0.041
**Residual 4**	0.92	0.086	0.057	-0.001	-0.002	0.002
**Scalar 5**	0.93	0.090	0.046	-	-	-
**Covariance 3**	-	-	-	-	-	-
**Covariance 4**	0.93	0.087	0.048	-0.001	0.003	-0.002

CFI = Comparative Fit Index; RMSEA = Root Mean Square Error of Approximation; SRMR = standardized root mean square residual; Δ (CFI, RMSEA, SRMR) = change in index value compared to baseline model. Models: Configural = no constraints across gender (1) and age (2) groups; Metric = nested within the configural, constrained regression weights of the indicators onto nuisance (1, 4) and general (2, 3, 5) latent factors; Scalar = nested within the full metric, constrained intercepts of the indicators (1, 3) and the nuisance factors (2, 4, 5); Partial scalar 1 = constrained intercepts of the indicators except for sexual distress; Residual = nested within the highest level of invariance established, constrained residual variance of the indicators (1, 3) and the nuisance factors (2, 4); Covariance = nested within the highest level of invariance established, constrained residual covariance of the indicators (1, 3) and the nuisance factors (2, 4); Means 1 = nested within the full scalar, constrained latent means across age groups.

## Discussion

In a non-clinical sample of 13024 Finnish adults, we expanded the Internalizing-Externalizing meta-structure of psychopathology by including body-related symptoms, we examined disorder covariance across the latent dimensions and we explained observed gender and age variation in terms of diversity in these dimensions.

### Transdiagnostic factors and cross-disorder associations

We hypothesized that modeling measures of anger, aggressive behaviour, sexual distress, body image and eating attitudes, alongside more common measures of psychopathology, would reshape the Internalizing-Externalizing meta-structure and, potentially, demonstrate the existence of more than two broad constructs underlying the observed patterns of multi-morbidity. In support of this hypothesis, we found an independent dimension of body-related disorders, comprising measures of body image, eating attitudes and, once accounting for a common latent factor of psychopathology, sexual distress. We further demonstrated how body-related symptoms covary with Internalizing and Externalizing factors, without being explained by them, by analyzing their associations with other disorders.

Instead of exclusively determining which model fitted our data best, we employed unidimensional, multidimensional and hierarchical conceptualizations to interpret cross-disorder associations. Certain associations remained stable across models. A strong within-factor relationship was observed between measures of alcohol use and psychopathy in both the two-factor model and the orthogonal bifactor model. A cross-factor relationship was consistently observed between Body and an Externalizing measure, trait anger. Part of the variance in this measure was explained by the Body factor in the correlated three-factor model, and its residual variance was associated with eating attitudes in the correlated two-factor model. These constant connections are in line with the high rates of comorbidity frequently observed between psychopathy and substance use disorders [82] as well as between eating disorders subtypes and anger, whether managed with releasing or inhibiting strategies [83, 84]. We also observed how associations changed once accounting for a general “P” factor. Sexual distress was initially conceived as an indicator of Internalizing, with Body explaining a small portion of its variance, but became more strongly associated with Body in bifactor modeling. The “P” factor explained a larger amount of variance in trait anger and sexual distress, compared to their specific factors (in the orthogonal bifactor model) and, interestingly, accounted for the relationship of Body with the other types of psychopathology (in the oblique bifactor model). Overall, the “P” factor seemed to capture a “general distress” facet of our indicators. We speculate that common negative affect may be differently expressed, for instance in the form of low tolerance for frustration or high preoccupation with intimacy or self- image, so that diverse diagnosable outcomes may arise.

### Gender- and age-specific psychopathology

Lastly, we predicted that differences in clinical presentation across men and women, as well as across generations, would be explained by group differences in the mean levels of the latent factors. To our knowledge, this is the first study to date where multi-group measurement invariance of the bifactor model of psychopathology was conducted. Differences in the observed means were in the direction of women’s higher standing on the internalizing, body-related and trait anger indicators. Men showed higher levels of the remaining externalizing pathologies. Measurement invariance tests revealed substantial gender differences in factor intercepts that precluded reliable latent means comparisons. Even though the strength of the relations between the measured variables and the latent constructs was homogeneous (metric invariance), group differences in the intercepts might have determined differences in the observed means, regardless of differences in the latent means [81]. This result speaks against gender invariance of the bifactor model psychopathology, at least when body-related disorders are included. Given the well documented sex differences in prevalence and severity of disordered eating, body dissatisfaction and sexual dysfunctions [85, 86], we further emphasize the need for a gender-specific approach to research and treatment of these pathologies. On the other hand, full invariance was established across generations and small to moderate differences were measured on the specific and general latent constructs. Young adults (18–25) showed moderately higher levels on Internalizing and slightly higher levels on the Externalizing and “P” factors. Middle-aged individuals (33–49) showed significantly lower mean levels of Internalizing. These results fit well in the existing literature reporting lower negative affect and greater well-being with increased age [87]. Nevertheless, middle-aged adults had the highest standing on the Body construct. The possibility of an early onset of menopause, which has been repeatedly associated with body-related pathologies [88, 89], offers an explanation for some of the women in our sample, but certainly not all. The interpretation of this finding in men is even more debatable. Research on body image, eating behavior and sexual life in middle-aged non-clinical men is scarce and, in contrast with our results, it points towards greater body satisfaction in middle-aged men, who are thought to be less influenced by media-portrayed idealized body images, compared to young men [90]. Further research is therefore needed to clarify gender and age- specific factors contributing to body-related problems.

### Limitations

The main limitations we want to outline when drawing our conclusions reside in the sampling and assessment procedures adopted. Our sample comprised Finns only, hence we cannot conclude that our results would generalize to other genetic and cultural groups. Participants were twins and siblings of twins, a non-random sample of the general population, but research on external validity of twin and family studies overall support their representativeness [91, 92]. Although we have been able to correct for the nested nature of our data, we had to assume homogeneity of family dependency across twins and their siblings to perform factorial analyses. Participants’ responses were collected via self-report, rather than by clinically trained interviewers. Even so, self-report measures are not necessarily disadvantageous. Other assessment methods suffer from many of the same measurement artefacts and, interestingly, a recent study indicated that similar factor structures emerge when symptoms are collected via self-report and clinical ratings [93]. Most importantly, despite the variety of indicators available, we could not include all measures of psychopathology in our factor analysis. Among notable exclusions, psychotic and autism spectrum disorders seem to reflect unique factors [22, 94] but were precluded because of our data collection method. Other omitted disorders have been shown to primarily load onto Internalizing, such as social and specific phobias, panic and post-traumatic stress disorder [95] as well as onto Externalizing, like drug dependence and conduct disorder [23]. Further extension of the Internalizing-Externalizing meta-structure might therefore highlight novel spectra.

Nevertheless, the adopted methodology presents notable advantages. Firstly, we chose to measure psychopathology dimensionally, because artificial categories do not capture truly distinct facets of mental illness and are not supported by categorical etiologies [17]. Investigating latent transdiagnostic factors, underlying pathological patterns and possibly sharing biological markers, is vital to revolutionize psychiatric classification method towards a dimensional nosology and to ultimately improve psychological and pharmacological treatment [96]. Secondly, we could validate our results with alternative models applied in comorbidity research. We believe that evidence converging from multiple directions is more likely to reflect the true underlying structure of a phenomenon. Thirdly, we conducted measurement and structural invariance tests which explicitly verifies the ability of measurement models to capture the same construct across groups. This method is preferable to techniques predominantly employed in psychological research to compare groups, such as t-tests and analysis of variance, which only assume that individuals with identical observed scores have the same level of the underlying construct, regardless of group membership.

## Conclusions

The present study provides useful evidence for the development of empirically-derived disorder classification and treatment approaches. We demonstrated that body-related problems characterize a phenotypically distinct factor and that the relationship between this factor and other internalizing and externalizing symptoms may be understood in light of a general latent dimension of psychopathology. Moreover, we described an interconnection between anger and attitudes towards food, self-image and sexuality. We measured higher levels of each of these indicators in women, as well as higher levels of the Body construct in middle-aged individuals. Altogether, the present results motivate further research on the etiological and clinical implications of cross-disorder, gender- and age-specific patterns of body-related symptoms.
